# Leaders as the targets of workplace bullying - prevalence and outcomes

**DOI:** 10.1007/s00420-024-02066-y

**Published:** 2024-04-15

**Authors:** Morten Birkeland Nielsen, Mariama Cham Evensen, Sana Parveen, Live Bakke Finne

**Affiliations:** 1 National Institute of Occupational Health, PB 8149 Dep, Oslo, N-0033 Norway; 2 Department of Psychosocial Science, University of Bergen, Bergen, Norway

**Keywords:** Mistreatment, Aggression, Management, Supervisors, Health

## Abstract

**Purpose:**

Bullying of leaders is an underexplored topic in organizational research. To fill this knowledge gap, the aims of this study were to determine the prevalence of bullying of leaders and to examine whether holding a formal leadership position influences the relationships between exposure to bullying and the outcomes job satisfaction and depression.

**Methods:**

Data from two separate surveys were employed: (1) A cross-sectional occupation specific sample comprising 678 Norwegian child welfare social workers; (2) A nationally representative probability sample of 1,608 Norwegian employees with two time-points (6 months’ time-lag).

**Results:**

Analyzing multiple indicators of workplace bullying, holding a formal leadership position had no impact on the initial risk of being bullied. Analyses of prospective data showed that leaders report a somewhat stronger increase in levels of bullying over time compared to non-leaders, although the effect size was small. With exception of a small buffering effect on the cross-sectional association between exposure to bullying behaviors and job satisfaction in the second sample, holding a leadership position had no effect on the strength of the association between bullying and outcomes.

**Conclusion:**

The findings show that leaders have the same risk of being bullied and are influenced by bullying in roughly the same manner as non-leaders. Organizational measures and interventions against bullying should therefore consider leaders as a risk group in line with other employees.

The concept “workplace bullying” refers to situations “*where an employee repeatedly and over a prolonged time period is exposed to harassing behavior from one or more colleagues and where the targeted person is unable to defend him−/herself against this systematic mistreatment*” (Einarsen 2005). Hence, as a phenomenon, workplace bullying is a process that involves two distinct, but overlapping, phases. The first is the systematic exposure to harassing behaviors from others at the workplace (exposure phase), while the second is the cognitive experience of being unable to handle this exposure, i.e., being in power imbalance (victimization phase). Estimates show that as many as 15% of employees at a global basis are exposed to workplace bullying behaviors, whereas 11% perceive themselves as victims of bullying (Nielsen et al. 2010). An extensive body of research has documented that being a target of bullying is significantly associated with a range of negative outcomes, including reduced mental and somatic health and well-being (Nielsen and Einarsen 2012; Verkuil et al. 2015), increased sickness absence (Nielsen et al. 2016), disability (Clausen et al. 2019; Nielsen et al. 2017a), and risk of suicide (Conway et al. 2022). As for the relative impact of bullying compared to other work exposures, studies suggest that being exposed to bullying is more detrimental than experiencing well-known stressors such as high job demands, role stressors, job insecurity, low social support, and lack of effort reward-balance (Niedhammer et al. 2012; Schutte et al. 2014).

Considering the prevalence and severity of workplace bullying, knowledge about which employees that have the highest risk of being bullied is an important starting point for further addressing and reducing bullying at workplaces as well as for guiding theoretical models and research hypotheses on this subject. However, although several previous studies have examined potential groups at risk (Zapf et al. 2011), little is known about whether having a formal leadership position at the workplace influences the likelihood of being bullied as well as the outcomes following the exposure to bullying (Branch et al. 2021). Rather, most studies on bullying and leadership have addressed the leader as the perpetrator of bullying or the role of leadership regarding the occurrence, predictors, and outcomes of bullying (e.g., Nielsen 2013; Stapinski et al. 2023). The overarching aim of this study was therefore to compare the prevalence of bullying of leaders with non-leaders using three different indicators of bullying (exposure to bullying behaviors, victimization, and perceived power imbalance) and to determine whether holding a formal leadership position at a workplace influences the magnitude of the outcomes following exposure to bullying behaviors.

To be able to fill the knowledge gap regarding leadership position and bullying, we first need to understand why and how holding a formal leadership position could have any impact on the risk of being bullied, as well as the outcomes following bullying. We will argue that holding a leadership position, theoretically, can both lessen and strengthen the risk of being bullied, as well as the association between exposure to bullying and a given outcome. The main reason is that in the context of workplace bullying, the legal authority and power associated with the leadership position can be a double-edged sword. In the following, we will elaborate on this contrasting effect.

## Leadership position and risk of bullying

As described in the abovementioned definition of workplace bullying, a perceived power imbalance between the bullied and the bully is highlighted as a key definitional criterion. That is, to label an event as bullying, the targets need to consider themselves as inferior to the perpetrators and therefore unable to retaliate or defend themselves against the experienced mistreatment (Einarsen 1999). This power imbalance could be either due to the formal power of organizational position or due to the informal power, such as, social support, knowledge and experience (Einarsen 2000). In an organizational context, a leader is someone who is formally in charge of organizing, guiding, and managing others, and being in a formal leadership position therefore includes a legitimate right to exercise control and influence (French and Raven 1959). Hence, intuitively, it seems reasonable to expect that leaders should have a reduced risk of experiencing a power imbalance with a subordinate colleague as the authority associated with the leadership position should provide the leader with effective means to retaliate and even stop the mistreatment. Extending this line of reasoning, it seems likely that bullying should have less impact on those holding a leadership position. That is, assuming that the formal authority related to the leadership position reduces the likelihood of being in power imbalance, a target of bullying holding a leadership position will be more likely to handle the mistreatment, or even retaliate against the mistreatment, and the exposure should have less impact on their health, well-being, and work ability.

However, in contrast to the above arguments, there are also reasons to expect that those holding a formal leadership position could lead to an increased risk of being targeted by, and react more strongly to, bullying. Being a leader is often recognized as the most exposed position in a team or work group since the leader is responsible for decision making and resource allocation, as well as balancing the prioritization of the organization’s goal achievements against the requirements of the subordinates. Hence, through their responsibilities, leaders will have a strong impact on the occurrence of work stressors such as high work pressure, role conflict, role ambiguity, internal competition, and disagreements with others at the workplace (Hauge et al. 2007; Van den Brande et al. 2016). In line with the “work environment hypothesis” which states that a work environment characterized by high levels of job demands creates a fertile ground for social tension which then may escalate into workplace bullying if not properly managed (Leymann, 1996), the experience of incompatible demands and expectations around roles, tasks and responsibilities may create frustration and stress within a work group, especially in connection to rights, obligations, privileges and positions. Considering that the leader has a formal responsibility for the working environment, dissatisfied employees may blame the leader for their working conditions, and thereby voice their dissatisfaction through aggression towards the immediate leader (Branch et al. 2021; Tuckey et al. 2024). Supporting this argument, it has been proposed that isolated managers who have lost the support of their colleagues would be especially vulnerable to upwards bullying (Zapf and Einarsen 2011). This suggests that bullying of leaders represents a particular type of systemic disfunction that has different root causes and power dynamics relative to other forms of bullying (Tuckey et al. 2024). That is, since bullying, by definition, involves a power imbalance between target and perpetrator (Nielsen et al. 2022), leaders are only able to be bullied by subordinate staff when their legitimate position power is undermined (Branch et al. 2021). Consequently, assessing perceived power imbalance is a requirement for understanding bullying of leaders.

Regarding how holding a formal leadership position can strengthen the impact of bullying on outcomes, previous research indicates that being in power balance with the perpetrator has a paradoxical role in that it is more difficult for a target in more power or in a balanced power relation to handle and withstand the exposure to bullying behaviors (Nielsen et al. 2022). Specifically, building on theories on cognitive dissonance, it is assumed that for those who expect to be in power balance with the perpetrator, the exposure to bullying behaviors could lead to an incongruity between their self-perceptions of being able to withstand bullying, and how they in reality are being treated by the bully (Nielsen et al. 2017b). That is, being exposed to bullying behaviors is unexpected and unanticipated and will thereby create a pervasive feeling of dissonance in a leader if he/she is exposed to bullying at the workplace. This additional experience of strain could subsequently amplify any health and well-being consequences of the bullying (Nielsen et al. 2022).

Taken together, the relation between holding a formal leadership position at a workplace and risk of being exposed to bullying is unclear. In addition, there is an important knowledge gap regarding how bullying impacts leaders as it can be argued that holding a leadership position can both amplify and attenuate the effects of bullying on the health and well-being of those exposed. To add to the understanding of how holding a leadership position relates to power imbalance, risk of bullying, and the outcomes of bullying, this two-sample study will answer the following research questions (RQ):

### RQ1

*Are employees with a formal leadership position at a higher or lower risk of experiencing (a) exposure to bullying behaviors, (b) victimization, and (c) power imbalance with perpetrator compared to employees without leadership responsibility?*

### RQ2

*Does holding a formal leadership position weaken or strengthen the magnitude of the outcomes following exposure to bullying behaviors?*

To answer the above research questions and to secure the internal and external validity of the findings, this study will use two different samples of Norwegian employees. The first is a cross-sectional probability sample from a single occupational group. The second sample is a two-wave a nationally representative probability sample of workers representative of all occupational groups.

## Methods

### Sample 1. Child service social workers

#### Procedure and sample

Sample 1 were collected as part of the “Oslo Workplace Aggression Survey” (OWAS), a collaborative project between the National Institute of Occupational Health in Norway (STAMI) and the vice mayor of education and child services in Oslo municipality. The survey was conducted electronically in March 2020. All employees (*N* = 1,264) working full or part time in the child welfare service in Oslo municipality received an email with an invitation to participate in a survey in which the employees were asked to fill in an anonymous self-reporting questionnaire. As all employees in the organization were invited to participate, the sampling approach can be described as a probability procedure. To ensure anonymity, the researchers were not informed about any identifying information. The Regional Committees for Medical and Health Research Ethics in Norway (REC South East) approved the project, including the procedure for informed consent (project number 28,496). In line with the General Data Protection Regulation (GDPR), STAMI acquired permission from the Norwegian Agency for Shared Services in Education and Research (SIKT; approval: 226,309) to process the personal data in this project for research purposes.

A total of 678 questionnaires were returned, yielding a response rate of 53.6%. The sample consisted of 74.4% women and 25.6% men. The mean age was 39 years (SD = 10.91). A total of 82.4% worked in a full-time position, 10.4% in a part-time position, while 6.6% were on-call staff. 0.6% were on temporary leave. Altogether 16.6% of the respondents had some sort of formal leadership responsibility.

#### Inventories

Formal position as a supervisor was assessed with a single item: “Do you have leadership responsibility at your workplace?” Response categories were: “no”, “yes, with professional responsibilities”, “yes with personnel responsibilities”, and “yes with professional and personnel responsibilities”. To retain statistical power, the three latter categories were classified into a single “Formal leadership” category.

Victimization from workplace bullying was measured with the single item self-labeling method (Einarsen and Skogstad 1996). After being presented with the following definition: “Bullying (harassment, badgering, niggling, freezing out, offending someone) is a problem in some workplaces and for some workers. To label something bullying it must occur repeatedly over a period of time, and the person confronted has to have difficulties defending himself/herself. It is not bullying if two parties of approximately equal “strength” are in conflict or the incident is an isolated event,” respondents were asked “Have you been subjected to bullying at the workplace during the last 6 months?”. The response categories were “no,” “rarely,” “now and then,” “once a week,” and “several times a week.” In line with previous research using this indicator of exposure to bullying (Nielsen et al. 2021), positive responses, i.e., “rarely” to “several times a week” were recoded into a single “self-labeling” category. Due to the relatively low prevalence of self-labeled bullying in the current study it was not possible to set a stricter threshold for self-labeling. Note that the reported prevalence rate is in line with previous research from Norway (Nielsen et al. 2009).

The nine-items Short Negative Acts Questionnaire (S-NAQ) was used to measure exposure to specific bullying behaviors at the workplace (Einarsen et al. 2009; Notelaers et al. 2018). The respondents were asked how often they had experienced negative behaviors such as being withheld information, being excluded or humiliated and being given unmanageable workloads, during the last 6 months, with response categories on a 5-point frequency scale ranging from 1 = “never,” 2 = “occasionally,” 3 = “monthly,” 4 = “weekly,” to 5 = “daily”. The S-NAQ had a Cronbach’s alpha value of 0.85 in the present study.

As the S-NAQ does not measure power imbalance explicitly, power relation between target and perpetrator as seen from the target’s perspective was assessed with a three-item scale developed as an add-on to the S-NAQ (Nielsen et al. 2022). An example item is “If you have been exposed to one or more of the behaviors in the list above, did you experience it as difficult to defend yourself against this treatment?”. Response alternatives were “never,” “sometimes,” “once in a while,” “often,” and “every time.” Higher scores indicate that the target is in power imbalance with the perpetrator. Cronbach’s alpha for the power relation scale was 0.91.

Job satisfaction was measured with the short version of The Job Satisfaction Scale (Brayfield and Rothe 1951) as presented by Hetland et al. (2008). Example items are: “I feel fairly satisfied with my present job” and “most days I am enthusiastic about my work”. Responses were given on a 5-point Likert scale where 1= “strongly disagree’” and 5= “strongly agree”. Cronbach alpha in study was 0.87.

Symptoms of depression during the last week were measured by 10 items from the Hopkins Symptom Checklist (HSCL-25). The HSCL is a valid and reliable self-administered instrument measuring mental distress (anxiety, depression) in population surveys (Derogatis et al. 1974). Earlier comparisons show that shorter versions perform as well as the more extensive versions of the inventory (Strand et al. 2003). Responses were given on a four-point scale, ranging from “1 = not at all” to “4 = extremely”. Example items are “Feeling no interest in things” and “Feeling hopeless about the future”. Cronbach’s alpha for this scale was 0.87.

### Sample 2. Nationally representative probability sample

#### Sample and procedure

This two-wave prospective study used a nationally probability sample of 5,000 employees that was drawn from The Norwegian Central Employee Register by Statistics Norway. This is the official register of all Norwegian employees, as reported by employers. The inclusion criteria were adult employees employed in a Norwegian enterprise. In Norway, most finish their primary and secondary education the year they turn 18, and a worker may start drawing retirement pension from the month after turning 62 years. To obtain a sample of adult employees and to be able to retain the full sample at both baseline and follow-up, only employees born between 1955 and 1997 were invited to the survey. Questionnaires were distributed in spring 2015. The overall response rate was 32%. In total, 1,608 of the questionnaires returned at baseline were satisfactorily completed. The survey and procedure for informed consent had approval from the “Regional Committee for Medical Research Ethics for Eastern Norway” (approval 2014/1725). All respondents provided informed consent to participate in the survey and the responses were treated anonymously. At T1, the study sample consisted of more women (52%) than men (48%). The mean age was 45.19 (SD = 10.04) years with a range from 21 to 61. In total, 53% were married, 26% were common-law partners, 14% were unmarried, and 7% were widowed, separated, or divorced. Altogether 9% had primary school as the highest educational level, 31% had high school, 32% had lower-level university, while 28% were graduates or postgraduates. The average job tenure was 11.3 years. 36% of the participants had a leadership role that included personnel responsibilities.

Using the same procedure and questionnaire as the T1-assessment, the follow-up data (T2) was collected six months later. The time-lag was based on previous prospective studies documenting longitudinal associations between work stressors and turnover intentions with similar time-lags (Kelloway et al. 1999; Nohe and Sonntag 2014). This period seems long enough to measure possible changes in individual scores, and not too long regarding non-response. To be able to examine changes in study variables over time, *only those who participated at the baseline assessment were invited to participate at T2*. Altogether 1149 respondents (72%) responded at T2. Attrition analyses showed that the T2 respondents (M = 46.75; SD = 18.85) were significantly (*t =* 4.57; *df* = 1603, *p <* .001) older than non-respondents (M = 42.49; SD = 10.45). There were no differences in the distribution of gender (X2 = 1.31; df = 1; *p* > .05) or formal leadership responsibility (X2 = 1.94; df = 1; *p* > .05). There were no significant differences in the main study variables at T1 between responders and non-responders at T2. These findings indicate that the study cohort is representative for the overall sample.

#### Inventories

Formal position as a supervisor was assessed by a single item question asking, “Do you have position as a supervisor?” Response categories were “no” and “yes.” Power imbalance was not assessed in Study 2. Otherwise, Study 2 used the same measurement instruments as included in Study 1. Cronbach’s alpha values were satisfactory for the S-NAQ (T1:0.81; T2: 0.87), Job satisfaction scale (T1: 0.81; T2: 0.80), and HSCL_Depression (T1: 0.86; T2: 0.86).

### Statistical analyses

Statistical analyses were conducted using IBM SPSS Statistics 28.01 and the Process 4.2 macro script for SPSS (Hayes 2013). The prevalence of self-labeled exposure to bullying use was assessed with frequency analyses and chi-square tests. Differences in the levels of exposure to bullying behavior and power imbalance between formal leaders and non-leaders were tested using t-tests and supplementary regression analyses that adjusted for age and gender (Study 1 and 2) and number of employees at worksite (Study 2). Differences in the magnitude of the association between exposure to bullying behaviors and outcome variables were determined by means of two-way interaction analyses in cross-sectional and prospective data. Continuous scale variables were centered in the analyses. Level of significance was *p* < .05.

## Results

### Study 1

A series of analyses of the different indicators of workplace bullying were conducted to determine whether holding a formal leadership position influences the risk of being exposed to bullying. Using self-labeled victimization as the dependent variable, a chi-square test showed no significant differences (X2 = 0.80; df = 1; *p* > .05) in experienced bullying among leaders (5.5%) and non-leaders (5%). A t-test of differences between leaders (M = 1.18; SD = 0.35) and non-leaders (M = 1.15; SD = 0.28) found no differences in levels of exposure to bullying behaviors (t=-0.67; df = 563; *p* > .05). Among those who reported exposure to at least one instance of bullying behaviors, there were no significant differences between leaders (M = 1.55; SD = 0.87) and non-leaders (M = 1.57; SD = 0.87) in levels of perceived power imbalance with the perpetrator (t = 0.17; df = 355; *p* > .05). Follow-up regression analyses with exposure to bullying behavior and power imbalance, respectively, as outcomes and with age and gender as control variables replicated the findings from the above t-tests.

Regression-based interaction analyses examine whether holding a leadership position may lessen or strengthen the association between exposure to bullying and (a) depression and (b) job satisfaction. The main findings from the interaction analyses are presented in Table 1. The findings show that exposure to workplace bullying was significantly associated with both outcomes. Holding a leadership position had no direct effects on depression and job satisfaction, as well as no moderating effects on the associations between exposure to bullying behaviors and the two outcome variables. Hence, the finding that leaders and non-leaders report equally strong relations between exposure to bullying and outcomes indicates that holding a leadership position neither strengthens nor lessens the associations in question.

**Table 1:** The impact of formal leadership position on outcomes following exposure to workplace bullying in Study 1 (*N* = 565)

*Predictor variable*	Outcome						
	Depression (R2 = 0.10)				Job satisfaction (R2 = 0.11)		
	B	SE B	95% CI B		B	SE B	95% CI B
Age	− 0.00	0.00	− 0.01 – 0.00		0.01	0.00	0.00 – 0.01
Gender	0.11*	0.05	0.02 – 0.20		− 0.14**	0.08	− 0.30 – − 0.03
Bullying behaviors (BB)	0.43***	0.07	0.30 – 0.55		− 0.77***	0.12	− 0.99 – − 0.54
Leadership position (LP)	− 0.06	0.06	− 0.17 – 0.05		0.15	0.10	− 0.04 – 0.34
Interaction BB*LP	− 0.04	0.16	− 0.35 – 0.27		− 0.07	0.27	− 0.61 – 0.46

***p < .001; **p < .01; *p < .05

### Study 2

#### Cross-sectional analyses of baseline (T1) data

A chi-square test found no significant differences between leaders (10.9%) and non-leaders (10.9%) regarding self-labeled victimization to bullying at T1 (X2 = 0.99; df = 1; *p* > .05). In addition, no difference between leaders (M = 1.20; SD = 0.34) and non-leaders (M = 1.20; SD = 0.34) was established for levels of exposure to bullying behaviors at T1 (t=-0.49; df = 1577; *p* > .05). A regression analysis with exposure to bullying behavior at T1 as outcome variable, adjusted for age, gender, and number of employees at the workplace as control variables supported the findings from the t-test.

**Table 2:** Cross-sectional analyses of the impact of formal leadership position on outcomes at T1 following exposure to workplace bullying in Study 2 (*N* = 1540)

*Predictor variables (T1)*	Outcome						
	Depression T1 (R2 = 0.14)				Job satisfaction T1 (R2 = 0.11)		
	B	SE B	95% CI B		B	SE B	95% CI B
Age	− 0.00	0.00	− 0.00 – 0.00		0.01	0.00	0.00 – 0.01
Gender	0.10***	0.02	0.07 – 0.14		0.06	0.03	− 0.00 – 0.13
Number of employees at worksite	0.00	0.00	00 – 0.00		0.00	0.00	00 – 0.00
Bullying behaviors (BB)	0.39***	0.03	0.34 – 0.45		− 0.80***	0.05	− 0.90 – − 0.71
Leadership position (LP)	− 0.05**	0.02	− 0.09 – − 0.01		0.14***	0.04	0.07 – 0.21
Interaction BB*LP	− 0.09	0.06	− 0.20 – 0.02		0.26*	0.10	0.06 – 0.46

***p < .001; **p < .01; *p < .05

**Fig. 1:**
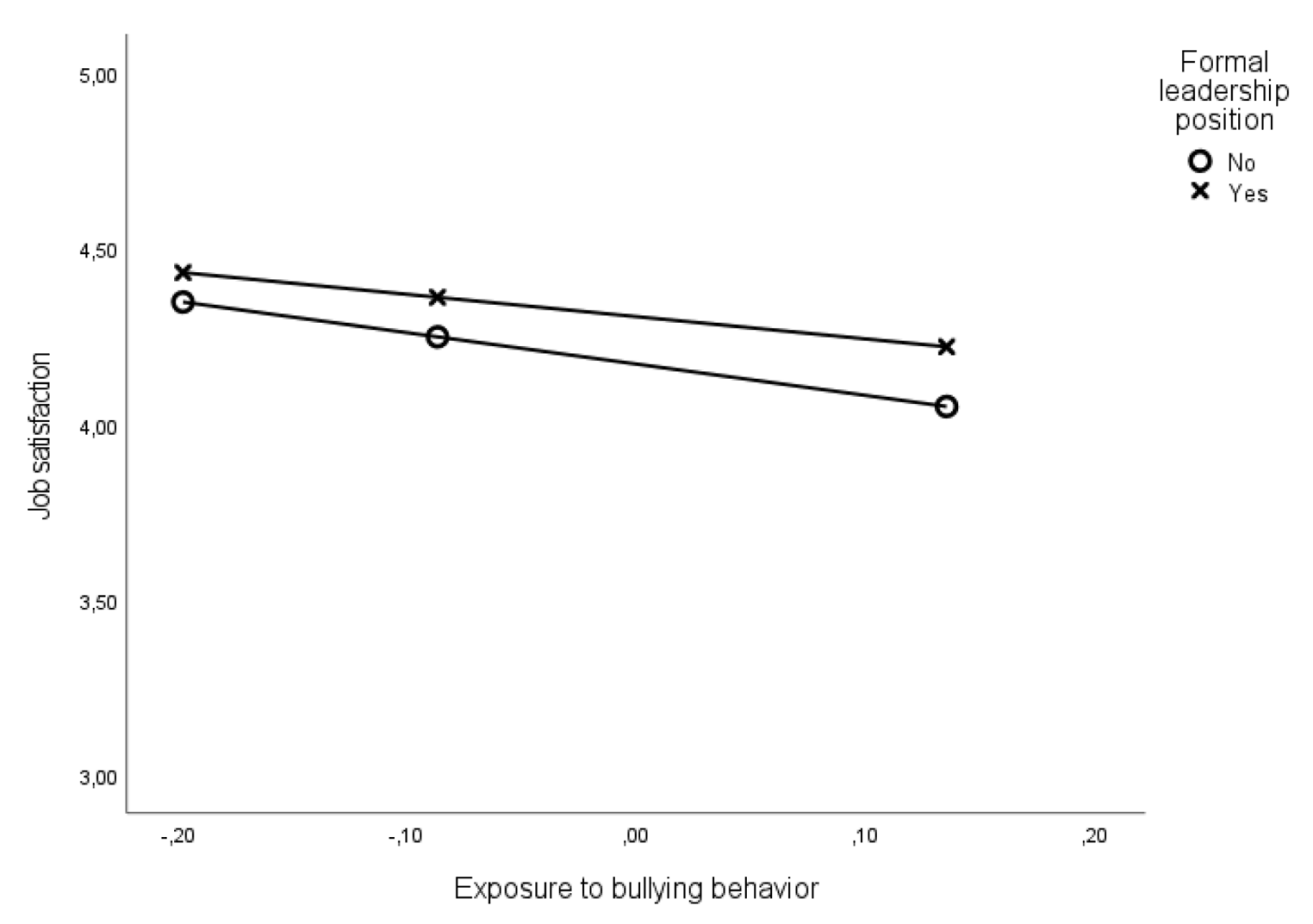
Cross-sectional associations between exposure to bullying behavior and job satisfaction at T1 for leaders and non-leaders in Study 2

As presented in Table 2, an interaction analysis showed no differences between leaders and non-leaders in the magnitude of the association between exposure to bullying behavior and levels of depressive symptoms at T1. However, a small significant difference between leaders and non-leaders was found for the strength of the association between exposure to bullying behaviors and job satisfaction at T1 (B = 0.26; *p* < .05). As displayed in Fig. 1, leaders (B = 0.64; *p* < .001) reported a somewhat weaker, albeit still significant, association between exposure to bullying behaviors and job satisfaction when compared to non-leaders (B = 0.89; *p* < .001).

#### Analyses of longitudinal data

**Fig. 2:**
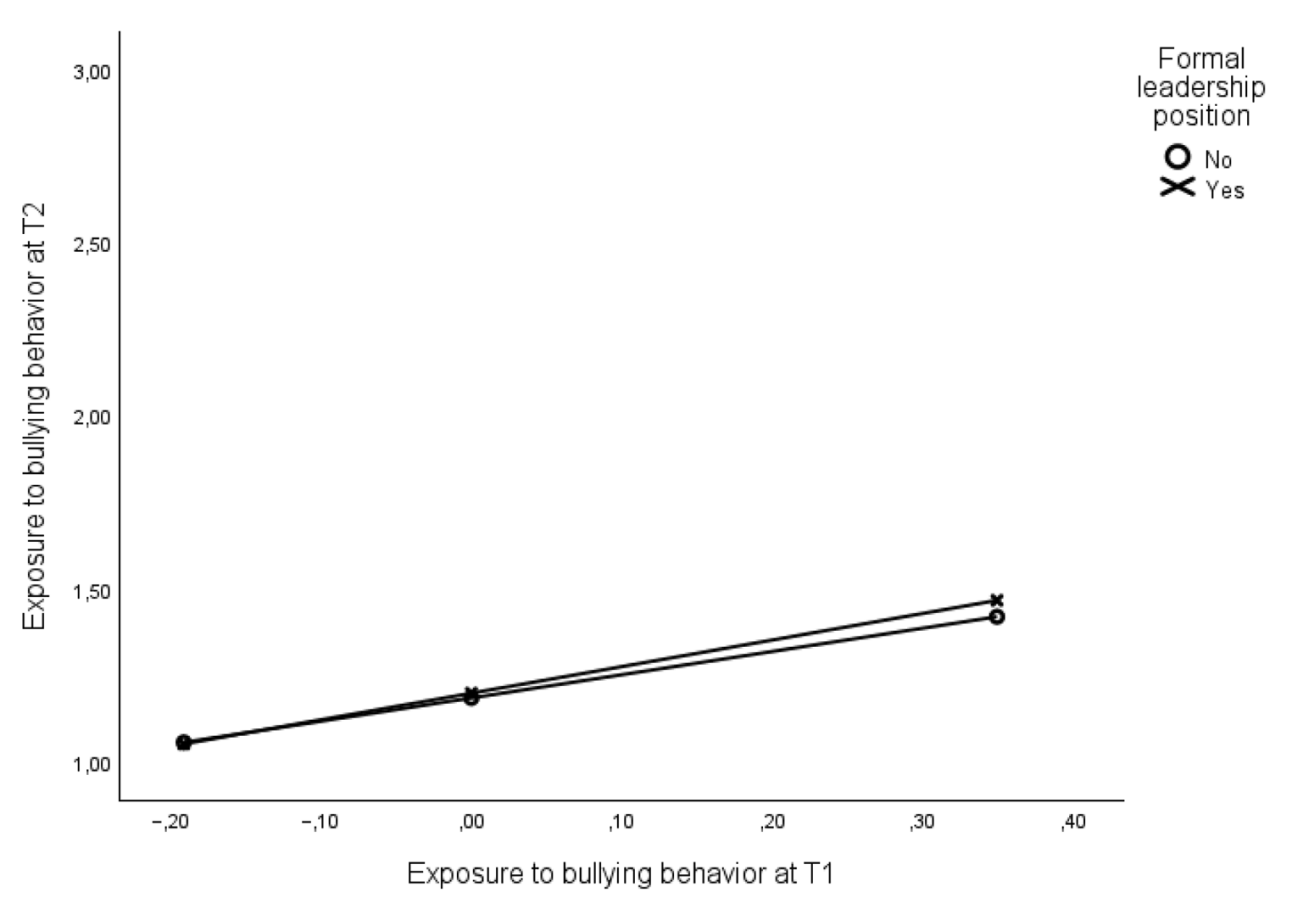
Associations between levels of bullying behavior at T1 with levels of bullying behavior at T2 for leaders and non-leaders in Study 2

Using levels of exposure to bullying behaviors at T2, adjusted for T1-levels, as the outcome variable, an interaction analysis with leadership position as the moderating variable examined whether holding a leadership position is associated with changes in levels of exposure to bullying behavior over time. Age, gender, and number of employees at the workplace were included as control variables. The results of the analyses showed that respondents holding a leadership position (B = 0.77; *p* < .001) reported a marginally, but significantly (B = 0.10; *p* < .05), stronger increase in levels of bullying behaviors when compared to respondents not holding a leadership position (B = 0.67; *p* < .001). As graphically displayed in Fig. 2, an inspection of the intercepts and slopes suggests that leaders exposed to bullying behavior experience a slightly stronger escalation in the exposure over time.

As displayed in Table 3, holding a leadership position had no impact on the associations between exposure to bullying behaviors and changes over time in levels of the two examined outcomes: depression and job satisfaction. That is, this indicates that leaders and non-leaders experience a similar magnitude in the outcomes following bullying.

**Table 3:** Longitudinal analyses of the impact of formal leadership position on changes in levels of outcomes following exposure to workplace bullying in Study 2 (*N* = 1096)

*Predictor variable*	Outcome at T2						
	Depression (R2 = 0.49)				Job satisfaction (R2 = 0.35)		
	B	SE B	95% CI B		B	SE B	95% CI B
Age	− 0.00	0.00	− 0.00 – 0.00		0.00	0.00	0.00 – 0.01
Gender	0.03	0.02	− 0.01 – 0.06		0.10**	0.04	0.02 – 0.17
Number of employees at worksite	0.00	0.00	00 – 0.00		0.00	0.00	00 – 0.00
Outcome at T1	0.72***	0.03	0.67 – 0.77		0.59***	0.03	0.53 – 0.64
Bullying behaviors (BB)	0.11***	0.03	0.05 – 0.16		− 0.21***	0.06	− 0.32 – − 0.10
Leadership position (LP)	0.01	0.02	− 0.03 – 0.05		0.02	0.04	− 0.06 – 0.10
Interaction BB*LP	− 0.03	0.05	− 0.13 – 0.08		-06	0.11	− 0.28 – 0.15

***p < .001; **p < .01; *p < .05

## Discussion

The overarching aims of the current study were to determine whether holding a formal leadership position influences the risk of being bullied at the workplace (measured as exposure to behaviors, victimization, and power imbalance with perpetrator) and whether holding such a position contributes to the magnitude of the outcomes associated with workplace bullying. In the introduction, we described two contrasting perspectives on how holding a formal leadership position could influence the risk of being exposed to bullying at the workplace as well as the outcomes following the exposure. The first perspective suggests that holding a leadership position is a protective factor that could decrease the risk of being exposed to bullying as well as reduce the potential impact of being bullied. The second perspective suggests that a leadership position is something that can increase the risk of being bullied and amplify the outcomes. In the following, we will summarize our findings, discuss how the findings inform these two perspectives, elaborate on the empirical and practical implications of the findings, discuss methodological limitations, and propose some venues for upcoming research on the topic.

### Summary of main findings

Based on data from two different samples of Norwegian employees, one occupation specific and one national probability, the results from the cross-sectional data indicate that holding a formal leadership position has no impact on the risk of being bullied. However, as shown by the results from the longitudinal data in the nationally representative probability sample, leaders report a somewhat stronger increase in levels of bullying over time. While this finding indicates that exposure to bullying among leaders subsequently becomes slightly more severe when compared to the cases of non-leaders, the effect size was quite small. The practical significance of this difference should therefore be questioned. With exception of a small buffering effect on the cross-sectional association between exposure to bullying behaviors and job satisfaction in Study 2, holding a leadership position had no effect on the strength of the association between bullying and outcomes. This indicates that leaders are influenced by bullying in more or less the same manner as non-leaders.

### Comparison with other studies and explanations for the findings

So how do our results relate to existing research? As for the overall prevalence rates of bullying, our findings on self-labeled victimization and exposure to bullying behavior correspond with a previous representative study of Norwegian employees (Nielsen et al. 2009), thus supporting the external validity of the data. However, the finding that leaders have an equal risk of being bullied as non-leaders goes against two previous studies. In a representative population study of Danish employees, it was established that managers/supervisors reported a significantly lower prevalence of bullying than unskilled workers (Ortega et al. 2009). Similarly, a study of Finnish business professionals found a somewhat lower prevalence of bullying among managers when compared to officials/clerks (Salin 2001). However, the latter study provided no formal significance test for the estimates, thus limiting inferences about actual differences in the prevalence for the two hierarchical groups. With such a limited knowledge base, it is difficult to draw any firm conclusions about leadership position as a risk factor for bullying, but taken together, it seems like holding a leadership position does at least not increase the risk of being exposed to bullying.

A noteworthy finding from the analyses of the longitudinal data in Study 2 was that those holding a formal leadership position reported a slightly stronger increase in exposure to bullying over time when compared to non-leaders. This indicates that although leaders have an equal risk of being bullied as others at the workplace, the level of exposure is likely to be experienced as slightly more escalated, i.e., more frequent, and severe, as time passes by. An explanation may be that the legal power status of the leaders makes them tougher to wear down as targets, and that this requires that the bully escalates the attempts at mistreatment. In line with this reasoning, it has been argued that to be able to undermine the legitimate power of the leader, perpetrators will move from covert and indirect behaviors to more overt and direct tactics due to a continuous power struggle between the parties (Branch et al. 2021).

The findings from Study 1 showed that bullied leaders and non-leaders reported similar levels of informal power imbalance with the perpetrator(s). Hence, despite their formal authority and assumed stronger ability to be able to defend themselves against mistreatment, leaders do perceive themselves just as inferior to the bully as do non-leaders. This indicates that the informal power dynamic between the bullied and the bully, and not the formal, is most important in cases of bullying. A practical implication of this finding is that organizations need to protect and take care of leaders in the same way as they support other employees. That is, just taking for granted that leaders can handle the mistreatment on their own can be a grave mistake, especially since leaders report similarly strong levels of depression following bullying as non-leaders. Rather, knowing that depression and other forms of psychological distress are main precursors to sickness absence and disability retirement, organizations need to have in place strategies for handling bullying does not exclude leaders. Previous research has shown that cultural factors, such as a strong climate for conflict management, may be especially valuable with regard to managing workplace bullying (Einarsen et al. 2018). Consequently, focusing on primary interventions, such as building a strong psychosocial safety climate may be the most effective way to prevent workplace bullying from occurring and harming employees (Bond et al. 2010).

### Methodological strengths and limitations

Several strengths and limitations should be considered in the interpretation of the present findings. As for strengths, the study includes two large probability samples and is based on previously validated instruments. Regarding limitations, the response rate of 32% in sample 2 is lower than the average rate in survey research (Baruch and Holtom 2008), and may limit the generalizability of the findings to some degree. However, nonresponse is a necessary but not sufficient condition for response bias. If the reason for nonresponse is uncorrelated with variables being analyzed, low response rates do not indicate response bias and lack of generalizability (Groves et al. 2004). Although the study used probability procedures, we were unable to assess whether the samples were fully representative of their overall population. This may potentially limit the external validity of the findings.

By using self-report measures, the study could be influenced by bias such as response-set tendencies and social desirability. Hence, it is possible that respondents may have underreported their actual levels on sensitive variables such as bullying and depression. In addition, the use of self-report measures may be vulnerable to common method variance (Podsakoff et al. 2003). Finally, the cross-sectional does not allow for any conclusions about such cause-and-effect relationships. For instance, previous research has shown that the association between bullying and depression is bidirectional, and it may therefore be that depression also increases the risk of being bullied (Verkuil et al. 2015). In order to determine the causal associations between the variables, future research should replicate this study using time-lagged data, as we have done in Study 2, although also such findings need to be interpreted with caution since also longitudinal designs have important limitations, including omission of third variables and being dependent upon using the optimal time-lag for detecting effects (Spector 2019). For instance, as the current study used a six-months lag between baseline and follow-up, it is possible that the responses were influenced by seasonal factors such as weather or hours of daylight (which varies extensively throughout the seasons in Norway).

It should be noted that the data for Sample 1 were collected at the outbreak of the Covid 19 pandemic in March 2020, and that this may have influenced the responses to the questionnaire, at least regarding the responses to the questions of job satisfaction and depression. As the questions about bullying were retrospective, asking about any exposure during the last six months before the survey, these are less likely to be influenced by the pandemic.

### Conclusions and suggestions for future research

Even though our findings point to some differences between leaders and non-leaders regarding exposure to bullying and the outcomes, the established effect sizes were small. Hence, based on our data there are few reasons to expect that the legitimate power related to the leadership position influences the risk of being bullied or the consequences associated with the exposure. That is, being exposed to bullying is just as problematic for leaders as it is for others at the workplace. However, as the current study only represents a single contribution to our understanding of bullying of leaders, more research is needed to validate our findings and to address further knowledge gaps. We suggest two ways forward: First, our findings need to be replicated in other countries and cultures. Norway is characterized by low scores on power distance (Warner-Søderholm 2012). As societies with low power distance tend to consider that all members are equal and have a low acceptance of differences in power and authority, it is likely that leader-member dynamics in Norway is different than in cultures with higher power-distance and that other findings would have been obtained in such cultures. Second, this study only examined whether participants holding a formal leadership position perceive themselves as bullied at the workplace without taking information about the formal status of the bully. Future research should therefore also ask about whether the bully is a subordinate, someone at an equal hierarchical level, or a superior and examine whether the hierarchical status of the bully have an impact on the consequences of the experienced bullying.

## Data Availability

The data that support the findings of this study are available from Statens Arbeidsmiljøinstitutt (STAMI), but restrictions apply to the availability of these data, which were used under license for the current study and so are not publicly available. The data are, however, available from the authors upon reasonable request and with the permission of STAMI.
